# Serum amylase levels are decreased in Chinese non-alcoholic fatty liver disease patients

**DOI:** 10.1186/1476-511X-13-185

**Published:** 2014-12-07

**Authors:** Jinmei Yao, Ying Zhao, Juanwen Zhang, Yani Hong, Huanle Lu, Jianping Wu

**Affiliations:** Department of Laboratory Medicine, The First Affiliated Hospital, College of Medicine, Zhejiang University, 79 QingChun Road, Hangzhou, 310003 Zhejiang Province People’s Republic of China

**Keywords:** NAFLD, Amylase, Metabolic syndrome, Fibrosis

## Abstract

**Background:**

Low serum amylase levels have been reported in patients with metabolic syndrome (MS), diabetes, and asymptomatic non-alcoholic fatty liver disease (NAFLD). However, no study has yet indicated the serum amylase levels in NAFLD with MS. The aim of the present study was to evaluate serum amylase levels in NAFLD patients with and without MS, and to explore a possible association between serum amylase levels with the components of MS and the degree of hepatic fibrosis in NAFLD patients.

**Methods:**

Our study included 713 NAFLD participants (180 females and 533 males) and 304 healthy control participants (110 females and 194 males). The diagnosis of NAFLD was based on ultrasonography, and advanced fibrosis was assessed by the FIB-4 index.

**Results:**

Serum amylase levels were significantly lower in NAFLD patients with MS compared with NAFLD patients without MS and healthy controls (42, 45, and 53 IU/L, respectively). The serum amylase levels of patients with elevated glucose, elevated triglycerides, and low high density lipoprotein cholesterol patients were significantly lower than in case of normal parameters (both p < 0.05). Multivariate logistic regression analysis showed that a relative serum amylase level increase was an independent factor predicting advanced fibrosis (FIB-4 ≥1.3) in NAFLD participants (OR: 1.840, 95% CI: 1.117-3.030, p=0.017).

**Conclusions:**

Compared with NAFLD patients without MS and healthy controls, serum amylase levels were significantly lower in NAFLD patients with MS. Moreover, a relative serum amylase increase may be an independent factor of more advanced hepatic fibrosis.

## Introduction

NAFLD is a clinico-pathological condition and corresponds to a disease spectrum encompassing simple steatosis, nonalcoholic steatohepatitis (NASH) with or without cirrhosis, and hepatocellular carcinoma (HCC) [1–5]. Approximately, 5% to 20% of patients with NAFLD develop NASH, which progresses to advanced fibrosis in 10% to 20% of cases and cirrhosis in nearly 5% of cases [3, 4, 6]. The prevalence of NAFLD in the general population in Europe is estimated to be 20% to 30% [7], and 12% to 24% in Asia [8]. In Shanghai, Guangdong, and Hong Kong (China) it has been reported to be 17%, 15%, and 16%, respectively [9–11].

NAFLD is closely associated with obesity, type 2 diabetes mellitus, metabolic syndrome (MS), insulin resistance, hypertension and dyslipidemia [12]. However, it is worth noting that nonalcoholic steatohepatitis also induces and enhances insulin resistance, leading to a vicious cycle [6]. Patients with NAFLD exhibit increased liver-related complications and mortality [6]. NAFLD has become a significant public health burden owing to hepatic and extrahepatic morbidity and mortality [2, 13].

The gold standard of hepatic fibrosis remains liver biopsy, but this technique is potentially risky and expensive [14]. Adams et al. [14] found that the FIB-4 index was the most appropriate indicator for advanced fibrosis prediction compared with other non-invasive fibrosis models. Likewise, Xun et al. found that the FIB-4 index, although slightly less accurate than liver biopsy, can be used to evaluate liver fibrosis in Chinese NAFLD patients [15].

Elevated serum amylase remains the most widely used biochemical test for the diagnosis of acute pancreatitis with serum amylase levels ≥ 3 times the normal upper limit [16, 17]. Low serum amylase has been reported in diffuse pancreas destruction and/or atrophic pancreas tissue [18–21]. Recently, other studies [22–26] have shown that lower serum amylase levels are associated with an increased prevalence for MS, diabetes, and NAFLD in asymptomatic adults and suggested that insulin resistance and fat accumulation may result in a decrease of serum amylase levels. Morever, Nakajima K et al. indicated that low serum amylase may be a marker for moderate or severe NAFLD [25].

Since NAFLD and MS can each lead to the decrease of serum amylase levels, we hypothesized that the combination of NAFLD and MS could further accentuate this decrease. Therefore, we aimed to explore the diagnostic value of serum amylase levels in the context of NAFLD with and without MS.

## Materials and methods

### Patients

#### Ethics statement

This study was approved by the ethics committee of the First Affiliated Hospital of the Medical College at Zhejiang University in China and was performed in accordance with the Helsinki Declaration. Written informed consent was obtained from each participant at the time of enrollment.

#### Inclusion and exclusion criteria

The diagnosis of MS was based on the Chinese Diabetes Society (CDS) classification when any three or more of the following five components were present [27]: (i) body mass index (BMI) ≥ 25 kg/m^2^ ; (ii) fasting plasma glucose (FPG) ≥ 6.1 mmol/L or taking anti-diabetic medications; (iii) blood pressure ≥ 140/90 mmHg or taking anti-hypertensive medications; (iv) triglycerides (TG) ≥ 1.7 mmol/L; and (v) high density lipoprotein cholesterol (HDL-c) < 0.9 mmol/L in males or < 1.0 mmol/L in females. Diabetes diagnosis was based on the 2010 International Expert Committee (IEC) and the American Diabetes Association (ADA) guidelines [28]. Diabetes mellitus was identified according to the following components: HbA1c ≥ 6.5%; random plasma glucose or 2-hour glucose ≥ 11.1 mmol/L; and fasting plasma glucose ≥ 7.0 mmol/dL. NAFLD was diagnosed according to the guidelines established for the diagnosis and treatment of NAFLD issued by the Chinese National Consensus Workshop on Nonalcoholic Fatty Liver Disease [29]. The diagnosis of NAFLD was based on ultrasonography finding of hepatic steatosis as diagnosed by characteristic echo patterns using a Toshiba Nemio 20 sonography machine with a 3.5-MHz probe (Toshiba, Tokyo, Japan). Hepatic steatosis was identified according to characteristics of the echo patterns, such as ultrasound beam attenuation, diffuse hyper echogenicity of the liver, and poor visualization of intra hepatic structures [30]. Patients with any of the following conditions in their medical history were excluded from the study: (i) alcohol consumption greater than 140 g/week for men and 70 g/week for women; (ii) viral hepatitis or autoimmune hepatitis; or (iii) hepatotoxic medications [31].

Healthy control participants were selected from clinically asymptomatic participants after exclusion of the following conditions: kidney, cardiovascular, liver, respiratory, and gynecologic diseases, impaired glucose tolerance, arterial hypertension, body mass index (BMI) ≥ 28 kg/m^2^ or ≤ 18.5 kg/m^2^, abnormal triglycerides (≥ 2.26 mmol/L) or total cholesterol (≥ 6.22 mmol/L), smoking (≥ 20 cigarettes per day) or drinking (≥ 30 g per day), the presence of pregnancy or lactation, surgery during the previous six months, acute or chronic infections, history of malignancy or drug intake within the previous two weeks.

Our study included 713 NAFLD participants (180 females [50.8 ± 13.4 years] and 533 males [44.5 ± 11.2 years]) and 304 healthy control participants (110 females [46.7 ± 11.5 years] and 194 males [46.0 ± 10.1 years]) who underwent a general health checkup in the context of the Health Care Centre at the First Affiliated Hospital of Medical College of Zhejiang University between September 2013 and February 2014. We divided the NAFLD participants into two groups: (1) NAFLD with MS (N = 300) and (2) NAFLD without MS (N = 413).

### Clinical and biochemical assessment

All study participants had a physical examination that included anthropometry, blood pressure measurement, and health habit inventory. Systolic blood pressure (SBP) and diastolic blood pressure (DBP) were measured using an automated sphygmomanometer with the subject in a sitting position. Body mass index (BMI) was calculated according to the following equation: body weight (kg) divided by the square of the height (m^2^). FIB-4 index was calculated according to the following equation: 

All study participants were subjected to the following biochemical determinations: serum amylase, alanine aminotransferase (ALT), aspartate aminotransferase (AST), triglyceride (TG), total cholesterol (TC), high density lipoprotein cholesterol (HDL-c), low density lipoprotein cholesterol (LDL-c), γ-glutamyltransferase ( γ-GT), FPG, creatinine (Cr), uric acid (UA), high sensitivity C reactive protein (hsCRP), platelet count (PLT), and glycated hemoglobin A1C (HbA1C) measurements.

All venous blood samples were obtained in the morning following a 12 h fast. Serum amylase, ALT, AST, TG, TC, HDL-c, LDL-c, γ -GT, and FPG were determined using a Hitachi DDP autoanalyzer (Hitachi Corp, Ibaragi, Japan). ALT, AST, TG, TC, γ -GT, and FPG levels were measured using Roche reagents (Roche Diagnostics, Indianapolis, USA). Serum amylase, HDL-c and LDL-c levels were measured using Shenshuoyoufu reagents (Shenshuoyoufu, Shanghai, China). HbA1c was determined on a Sysmex HbA1c analyzer G8 (Sysmex corporation, Kobe, Japan) using Sysmex reagents. PLT count was determined using a Sysmex XE-2100 automated hematology analyzer (Sysmex Corp, Kobe, Japan).

### Evaluation of liver fibrosis

No liver biopsy was performed. Instead, a non-invasive FIB-4 index of ≥1.3 was utilized to evaluate advanced fibrosis in NAFLD patients (defined as portal fibrosis with many septa and cirrhosis [14, 32]), according to studies by Xun et al. and Kim et al. [15, 33].

### Statistical analyses

Statistical analyses were performed using SPSS, version 16 (SPSS, Inc., Chicago, USA). Age, BMI, SBP, DBP, TC, HDL-c, LDL-c, FPG, and PLT count were reported as mean ± standard deviation (SD). Serum amylase, ALT, AST, γ -GT, TG, Cr, UA, HbA1C, HsCRP, and FIB-4 index were reported as the median and range. The differences among the multiple groups and between two groups (shown in Tables 1 and 2) were assessed using a one-way analysis of variance (ANOVA) and Kruskal-Walls H/Mann–Whitney U analysis, as appropriate. The differences in gender among the groups were compared using a Chi-squared test. Spearman correlation analysis was used to examine the correlation between serum amylase levels and clinical characteristics. Stepwise regression was used to select factors associated with the incidence of advanced fibrosis in NAFLD patients. Multivariate logistic regression was used to examine the associations between the amylase and the prevalence of advanced fibrosis in NAFLD patients. All statistical tests were two-tailed. p < 0.05 was considered statistically significant.

**Table 1:** **Baseline characteristics of study participants**

Variables	NAFLD (n = 713)		Healthy controls (n = 304)	P value
	Without MS (n = 413)	With MS (n = 300)		
Age (yr)	45.9 ± 12.1	46.3 ± 12.2	46.3 ± 10.6	0.874
Gender (female/male)	132/281#	48/252*	110/194	<0.001
BMI (kg/m^2^)	25.9 ± 2.7*#	26.8 ± 2.5*	22.8 ± 2.6	<0.001
SBP (mmHg)	131± 16*	132 ± 16*	120 ± 14	<0.001
DBP (mmHg)	80 ± 11*	81 ± 11*	78 ± 10	<0.001
ALT (IU/L)	26 (7–179)*#	33 (9–184)*	17 (6–39)	<0.001
AST (IU/L)	24 (12–103)*#	26 (14–122)*	20 (12–38)	<0.001
γ-GT (IU/L)	29 (8–248)*#	41 (7–236)*	18 (7–50)	<0.001
TG (mmol/L)	1.32 (0.34–3.74)*#	2.26 (0.63-21.05)*	0.97 (0.39–1.70)	<0.001
TC (mmol/L)	4.91 ± 0.91*#	5.06 ± 1.06*	4.51 ± 0.63	<0.001
HDL-c (mmol/L)	1.24 ± 0.34*#	1.01 ± 0.24*	1.27 ± 0.28	<0.001
LDL-c (mmol/L)	2.74 ± 0.57*#	2.62 ± 0.64*	2.49 ± 0.44	<0.001
FPG (mmol/L)	5.21 (3.82–7.52)*#	5.43 (4.23–16.46)*	4.76 (3.81–6.08)	<0.001
Cr (μmol/L)	73 (37–104) #	76 (34–108)*	70 (40–104)	<0.001
UA (μmol/L)	351 (66–548)*#	381 (146–547)*	309 (171–422)	<0.001
Amylase (IU/L)	45 (21–207)*#	42 (21–102)*	53 (7–122)	<0.001
HbA1C (%)	5.6 (4.2–6.9)*#	5.7 (4.6–12.5)*	5.5 (3.2–6.3)	<0.001
HsCRP (mg/L)	1.4 (0.3–19.4)*#	1.6 (0.3–15.2)*	0.9 (0.2–8.8)	<0.001
PLT (109/L)	233 ± 52*	231 ± 55*	214 ± 41	<0.001

Data are presented as mean ±SD or median (range).

P value: compared among three groups.

*p<0.05, compared with controls, #p<0.05, NAFLD without MS compared with NAFLD with MS.

**Table 2:** **Characteristics of NAFLD participants according to serum amylase quartile levels**

Variables	Total	Q1 (Lowest)	Q2	Q3	Q4 (Highest)	P value
Amylase (U/L)	44 (21–207)	33 (21–37)	41 (38–44)	49 (45–53)	62 (54–207)	
Age (yr)	46.1 ± 12.1	43.0 ± 11.0	45.5 ± 11.0	45.9 ± 11.9	50.5 ± 12.1	<0.001
Gender (female/male)	180/533	48/145	36/140	51/124	45/124	0.292
BMI (kg/m^2^)	26.2 ± 2.6	26.7 ± 3.0	26.3 ± 2.6	26.2 ± 2.5	25.6 ± 2.2	0.016
SBP (mmHg)	131 ± 16	132 ± 16	131 ± 15	131 ± 16	132 ± 17	0.804
DBP (mmHg)	80 ±11	80 ±11	81 ±11	80 ±11	80 ±10	0.460
ALT (IU/L)	29 (7–184)	30 (7–184)	33 (10–179)	28 (9–15)	26 (11–164)	0.011
AST (IU/L)	24 (12–122)	24 (12–103)	26 (15–122)	24 (13–55)	25 (15–101)	0.395
γ-GT (IU/L)	34 (7–248)	38 (8–236)	40 (9–248)	30 (11–145)	30 (7–139)	<0.001
TG (mmol/L)	1.67 (0.34–21.05)	1.73 (0.34–15.86)	1.70 (0.41–21.05)	1.55 (0.47–10.91)	1.57 (0.37–7.77)	0.040
TC (mmol/L)	4.97 ± 0.98	4.92 ± 0.90	5.07 ± 1.16	4.97 ± 0.95	4.92 ± 0.89	0.444
HDL-c (mmol/L)	1.14 ± 0.32	1.13 ± 0.45	1.13 ± 0.27	1.16 ± 0.26	1.15 ± 0.24	0.709
LDL-c (mmol/L)	2.69 ± 0.61	2.63 ± 0.57	2.73 ± 0.64	2.72 ± 0.62	2.69 ± 0.60	0.364
FPG (mmol/L)	5.29 (3.82–16.46)	5.51 (3.82–16.46)	5.25 (4.25–14.25)	5.18 (4.23–7.61)	5.23 (4.22–12.42)	<0.001
Cr (μmol/L)	74 (34–108)	73 (34–99)	73 (43–95)	72 (39–103)	75 (48–108)	<0.001
UA (μmol/L)	363 (66–548)	368 (66–548)	377 (146–547)	357 (195–536)	353 (157–509)	<0.001
Hba1C (%)	5.7 (4.2–12.5)	5.7 (4.8–10.5)	5.7 (4.2–12.5)	5.6 (4.7–7.8)	5.7 (4.6–11.5)	0.002
HsCRP (mg/L)	1.5 (0.3–19.4)	1.8 (0.4–16.5)	1.6 (0.3–19.0)	1.4 (0.4–19.4)	1.3 (0.3–17.0)	<0.001
PLT (109/L)	233 ± 53	241 ± 50	228 ± 51	233 ± 52	228 ± 59	0.058
FIB-4	0.89 (0.26–4.61)	0.77 (0.26–2.94)	0.90 (0.26–4.61)	0.92 (0.33–3.21)	1.03 (0.27–4.50)	<0.001
FIB-4 ≥ 1.3 (%)	156/713 (21.9)	27/193 (14.0)	37/176 (21.0)	37/175 (21.1)	55/169 (32.5)	<0.001
MS (%)	300/713 (42.1)	96/193 (49.7)	81/176 (46.0)	55/175 (31.4)	68/169 (40.2)	0.003

## Results

### Baseline characteristics of study participants

The baseline characteristics of the study participants are shown in Table 1. For the NAFLD with or without MS groups, BMI, SBP, DBP, ALT, AST, γ-GT , TG, TC, HDL-C, LDL-C, FPG, UA, amylase, HbA1C, HsCRP, PLT, and FIB-4 values were all statistically different as compared with the healthy control group (P<0.05 for all covariates). Moreover, for the NAFLD with MS group, BMI, ALT, AST, γ-GT, TG, TC, HDL-C, LDL-C, FPG, UA, amylase, HbA1C, HsCRP, and FIB-4 values were statistically different from the NAFLD group without MS (P<0.05).

**Figure 1:**
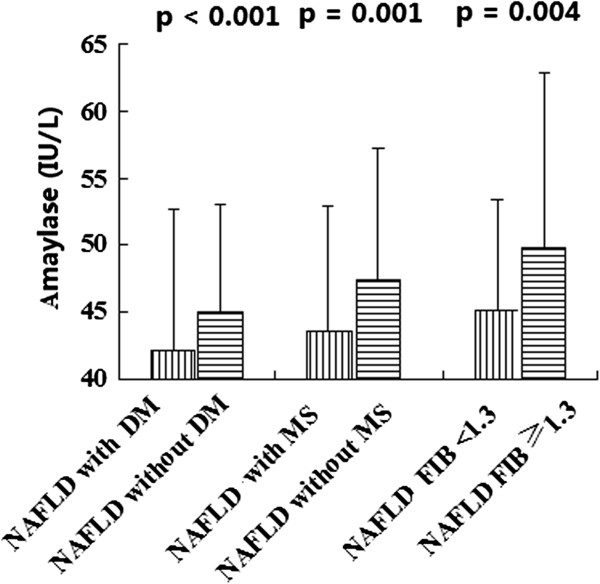
**Serum amylase levels in NAFLD patients with and without MS/DM, FIB-4 ≥1.3 and <1.3.**

**Figure 2:**
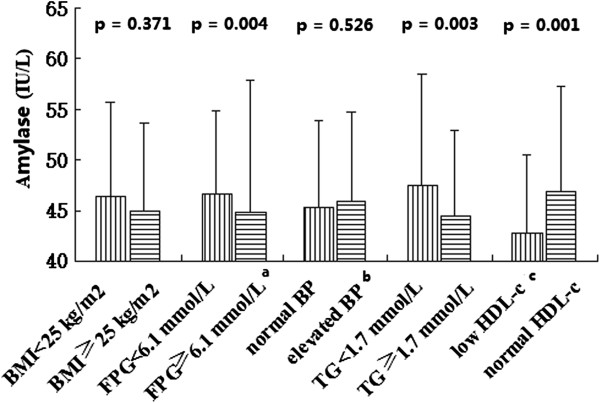
**Serum amylase levels of the five MS components in NAFLD. a**: fasting plasma glucose (FPG) ≥ 6.1 mmol/L or taking anti-diabetic medications, **b**: blood pressure ≥ 140/90 mmHg or taking anti-hypertensive medications, **c**: high density lipoprotein cholesterol (HDL-c) < 0.9 mmol/L in males < 1.0 mmol/L in females.

Serum amylase levels in NAFLD patients with MS/DM were significantly lower than those in NAFLD patients without MS/DM (both p < 0.05, Figure 1). Serum amylase levels of the five MS components in NAFLD with MS patients are shown in Figure 2. Serum amylase levels of patients with elevated glucose, elevated TG, and low HDL-c were significantly lower than in those with normal glucose, TG, and HDL-c (p < 0.05). However, serum amylase levels were not significantly different between normal and elevated BMI or elevated BP patients (p > 0.05).

### Characteristics of NAFLD participants according to the quartile of amylase levels

We divided NAFLD participants into four groups: Q1 (lowest), Q2, Q3, and Q4 (highest) according to the quartile of their serum amylase levels. Across increasing serum amylase quartiles, BMI, SBP, DBP, TG, FPG, and HsCRP levels were gradually decreased, while age, FIB-4 values and the incidence of FIB-4 ≥ 1.3 gradually increased. Age, BMI, SBP, DBP, ALT, γ-GT, TG, FPG, Cr, UA, HbA1C, HsCRP, FIB-4 values, and the incidence of FIB-4 ≥ 1.3 and MS were significantly different between the four groups.

### Correlation analysis between serum amylase levels and other variables

We found that the serum amylase levels in NAFLD participants were positively correlated with age, HDL-c, and FIB-4 (r > 0, p < 0.05) and negatively correlated with BMI, ALT, γ-GT, TG, FPG, UA, and HsCRP (r < 0, p < 0.05) (Table 3).

**Table 3:** **The correlation between serum amylase and other covariates**

Covariate	Correlation coefficient	P value
Age (yr)	0.212	<0.001
BMI (kg/m^2^)	−0.163	0.001
SBP	−0.024	0.541
DBP	−0.027	0.479
ALT (IU/L)	−0.101	0.007
AST (IU/L)	0.019	0.609
γ-GT (IU/L)	−0.158	<0.001
TG (mmol/L)	−0.108	0.004
TC (mmol/L)	−0.023	0.541
HDL-c (mmol/L)	0.105	0.005
LDL-c (mmol/L)	0.027	0.465
FPG (mmol/L)	−0.142	<0.001
Cr (μmol/L)	0.052	0.165
UA (μmol/L)	−0.101	0.007
Hba1C (%)	−0.058	0.122
HsCRP (mg/L)	−0.177	<0.001
PLT (109/L)	−0.116	0.002
FIB-4	0.233	<0.001

### Association of serum amylase and the incidence of advanced fibrosis in NAFLD participants

Serum amylase levels of the FIB-4 ≥ 1.3 group and the FIB-4 < 1.3 group were significantly different (p = 0.004, Figure 1). The results of an unadjusted and adjusted multivariate logistic regression analysis model (model 1–4) are presented in Table 4. Model 4, following adjustment for age, gender, BMI, history of MS, γ-GT, ALT, and HsCRP (p < 0.05 for all covariates), selected by stepwise regression, showed that serum amylase levels were independent factors predicting advanced fibrosis (FIB-4 ≥ 1.3) in NAFLD participants (OR: 1.840, 95% CI: 1.117-3.030, p = 0.017).

**Table 4:** **Odds ratios for advanced fibrosis in NAFLD participants according to serum amylase quartiles levels (Q4 vs. Q1)**

Model	Odds ratio (95% CI)	P value
Model 1	2.966 (2.199-4.002)	<0.001
Model 2	1.798 (1.103-2.930)	0.019
Model 3	1.782 (1.090-2.911)	0.021
Model 4	1.840 (1.117-3.030)	0.017

Odds ratios were determined using logistic regression analyses. Model 1: unadjusted; Model 2: adjusted for age, gender, and BMI; Model 3: adjusted for age, gender, BMI, and history of MS; Model 4: adjusted for age, gender, BMI, history of MS, γ-GT, ALT, and HsCRP.

## Discussion

The present cross-sectional study showed that NAFLD patients with MS had lower serum amylase levels than healthy controls and NAFLD patients without MS. The prevalence of MS in NAFLD patients was higher in the lower serum amylase levels group compared to the other three groups. Moreover, serum amylase levels of NAFLD patients with elevated FPG, elevated TG, or reduced HDL-c levels were lower than in NAFLD patients with normal FPG, TG, or HDL-c levels. In addition, serum amylase levels were increased, as an independent factor, in NAFLD patients with advanced liver fibrosis. These results provide evidence for a significant association between low serum amylase levels and NAFLD with MS.

The significant association between low serum amylase levels and NAFLD with MS suggest that MS and NAFLD may both contribute to the decrease in serum amylase levels. The potential mechanism accounting for the association between NAFLD and low serum amylase levels may be insulin resistance and fatty pancreas. Insulin resistance is known to be one of the key components of MS and it eventually leads to the development of type 2 diabetes, and NAFLD and NASH are tightly associated with insulin resistance [22–26]. In humans, a strong relationship exists between hepatic fat accumulation and whole-body insulin resistance. Morever, insulin resistance may enhance hepatic fat accumulation by increasing free fatty acid delivery and by stimulating the anabolic process due to hyperinsulinemia [34]. In 2014, Gruben et al. [35] found that hepatic lipid accumulation and inflammation could be the main drivers of hepatic insulin resistance. The association between lipid accumulation (such as diacylglycerol and TG) and hepatic insulin resistance has been observed in some animal models [36, 37]. Although the physiological liver maintains blood glucose homeostasis by gluconeogenesis and insulin inhibition, in hepatic insulin resistance, this inhibition is no longer effective [38]. Diacylglycerol is used for the formation of TG, a process catalyzed by the enzyme diacylglycerol acyltransferase 2 (Dgat2), and leading to protein kinase Cϵ activation (PKCϵ), which in turn results in hepatic insulin resistance [36, 39]. Reduction of diacylglycerol by down-regulation of Dgat2 can improve glucose intolerance and restore hepatic insulin signaling in mice [36]. Main inflammatory pathways, nuclear factor κB (NF-κB) activation regulated by the IKK2 or TNFR signaling cascade, c-Jun NH2-terminal kinase (JNK) activation, and Kupffer cell depletion are involved in the development of insulin resistance [40–42].

Some studies reported the associations between low serum amylase levels and MS, diabetes, NAFLD, cardiometabolic aspects, and insulin resistance after adjustement for relevant confounding factors [23, 25, 43, 44]. These associations may be related to insulin resistance and systemic ectopic fat deposition in the pancreas in asymptomatic adults [45]. In rat models, long-term exposure to a high-fat diet induced both interlobular and intralobular fat accumulation, pancreas fibrosis, and damaged the normal pancreatic architecture and islets [46]. In human studies, fatty pancreas is closely associated with increased insulin resistance, metabolic syndrome, and fatty liver [26]. NAFLD and MS have been reported to be associated with fatty pancreas [47, 48]. Some previous studies have indicated that fatty pancreas may lead to exocrine-endocrine dysfunction and to a loss of β-cell mass and function [25, 49], which may cause the decrease of serum amylase [25]. Wu et al. found that serum amylase values were significantly lower for the fatty pancreas as compared to normal pancreas [26]. Lee et al., and our previous study, also found that low serum amylase levels were associated with an increased prevalence of MS [22, 24]. Our results are consistent with the studies of Nakajima et al. [23, 25] who also suggested that low serum amylase levels may be associated with NAFLD and MS through insulin resistance and fatty pancreas.

Liver biopsy is the gold standard for determining the presence and degree of hepatic fibrosis in NAFLD patients, but liver biopsy has several shortcomings [50]. The identification of advanced fibrosis in NAFLD patients is of utmost important in clinical practice [15]. Therefore, non-invasive fibrosis models, such as APRI, BARD, Hepascore, Fibrotest, FIB4, AAR, and NIKEI, were developed for evaluating advanced fibrosis in NAFLD patients [14, 15, 51]. Xun et al., in China, showed that a FIB-4 index ≥ 1.3 for evaluating advanced fibrosis was better than the other non-invasive models and was suitable for evaluating advanced fibrosis in Chinese NAFLD patients [15]. Therefore, we chose to use this index for evaluating advanced fibrosis in our study.

NAFPD may lead to nonalcoholic steatopancreatitis (NASP), and pancreatic steatosis has also become increasingly and may affect the progression of NAFLD [48, 52, 53]. Patel et al. found that pancreatic fat content was lower in NAFLD patients who had advanced liver fibrosis as assessed by novel magnetic resonance imaging technology [53]. Although serum amylase levels in NAFLD patients were globally decreased, those NAFLD patients with advanced fibrosis had relatively higher serum amylase levels for less pancreatic fat content. Similar to our results, Nakajima et al. suggested that low serum amylase was associated with NAFLD independently of MS, diabetes and obesity and may be an independent marker for moderate/severe NAFLD in asymptomatic adults [25]. However, in their study, it appears difficult to evaluate the association between serum amylase and hepatic fibrosis since the study included a large number of non-obese individuals with only a small proportion having MS and diabetes, which may have resulted in a lower likelihood of advanced hepatic fibrosis.

Several limitations of our study should be mentioned. First, it is a cross-sectional observational study that cannot definitively comment on causality or temporal association between low serum amylase and NAFLD. Second, NAFLD diagnosis was based not on the gold standard of liver biopsy, but on ultrasonography, which may not be sensitive enough to detect mild steatosis. Third, for evaluating advanced fibrosis, we did not use liver biopsy but the surrogate FIB-4 index ≥ 1.3 [15]; it remains possible that some patients were inadequately classified. Finally, we only studied Chinese NAFLD patients and our results may not fully apply to other ethnic populations.
